# Evaluation of Anti-Inflammatory Effect of *Moringa oleifera* Lam. and *Cyanthillium cinereum* (Less) H. Rob. Lozenges in Volunteer Smokers

**DOI:** 10.3390/plants10071336

**Published:** 2021-06-30

**Authors:** Thitiya Luetragoon, Rungnapa Pankla Sranujit, Chanai Noysang, Yordhathai Thongsri, Pachuen Potup, Jukarin Somboonjun, Nucharee Maichandi, Nungruthai Suphrom, Supaporn Sangouam, Kanchana Usuwanthim

**Affiliations:** 1 Cellular and Molecular Immunology Research Unit, Faculty of Allied Health Sciences, Naresuan University, Phitsanulok 65000, Thailand; nok.hong.yok49@gmail.com (T.L.); yordhathai.k@gmail.com (Y.T.); pachuenp@nu.ac.th (P.P.); 2 Thai Traditional Medicine College, Rajamangala University of Technology Thanyaburi, Pathum Thani 12130, Thailand; rungnapa_s@rmutt.ac.th (R.P.S.); chanai_n@rmutt.ac.th (C.N.); 3 Wang Thong Hospital, Phitsanulok 65130, Thailand; shinomizu@hotmail.com (J.S.); nucharee_meddy@hotmail.com (N.M.); 4 Center of Excellence for Innovation in Chemistry, Department of Chemistry, Faculty of Science, Naresuan University, Phitsanulok 65000, Thailand; nungruthais@nu.ac.th; 5 Faculty of Dentistry, Naresuan University, Phitsanulok 65000, Thailand; supapornsa@nu.ac.th

**Keywords:** medical plant, astragalin, smoking, oral inflammation, gingivitis

## Abstract

Smokers have high plaque accumulation that initiates gingival inflammation and progresses to periodontitis. Thus, oral hygiene to control microbial plaque formation is an effective method of preventing gingivitis. Medicinal plants such as *Moringa oleifera* Lam. (MO) and *Cyanthillium cinereum* (Less.) H. Rob. (CC) have an anti-inflammatory effect that might improve oral health in smokers. This study evaluated the effect of MO leaf and CC extracts using MO lozenges and a combination of MO + CC lozenges on oral inflammation and gingivitis in volunteer smokers. Lozenges consisting of MO and CC extracts were developed and studied in vivo. The results showed that lozenges significantly reduced oral inflammation and gingivitis in volunteers. The gingival index (GI) of group III (MO + CC lozenges) significantly decreased, while the percentage decrease of oral inflammation in group II (MO lozenges) was significantly higher than the other groups. The percentage decrease of GI values in group II (MO lozenges) and group III (MO + CC lozenges) were significantly higher than the placebo group I. Our findings indicated that MO and MO + CC lozenges reduced oral inflammation and gingivitis and showed potential to improve oral health in smokers.

## 1. Introduction

Cigarette smoke contains a complex mixture of over 5300 chemical substances including nicotine and other carcinogens. Furthermore, cigarette smoke is a major source of free radicals including reactive oxygen species (ROS) and reactive nitrogen species (RNS) that cause oxidative damage of lipids, proteins, and deoxyribonucleic acid (DNA) [1,2]. Cigarette smoke induces inflammation by activation of IκB kinase (IKK), leading to the phosphorylation and proteasomal degradation of nuclear factor kappa B (NF-κB) inhibitor. This allows the translocation of NF-κB to the nucleus, where it regulates the expression of pro-inflammatory genes such as tumor necrosis factor (TNF)-α, interleukin (IL)-8, and cyclooxygenase (COX)-2 [3,4]. Therefore, smoking is a risk factor for various diseases, particularly oral diseases and oral mucosal lesions in smokers including smoker’s palate, smoker’s melanosis, coated tongue, oral candidiasis and dental caries, gingivitis, periodontal disease, implant failure, and oral cancer [5,6]. There is a strong correlation between smoking and progression of inflammatory diseases of gingival and periodontal tissues and mucosal surfaces. Periodontitis is more prevalent and more severe in smokers [7]. Relative risk of periodontal disease in smokers has been reported to be between 2.5- and 6-times higher compared to nonsmokers. Moreover, many studies found higher amounts of plaque accumulation in smokers [5]. Subgingival bacteria and dental plaque accumulation initiate gingival inflammation. If left untreated, this causes gingivitis and eventually progresses to periodontitis, with destruction of the underlying supporting tissue and alveolar bone [8,9].

Oral hygiene to control bacterial plaque biofilm formation is an effective method to prevent gingivitis [10]. There are various preventive treatments for gingivitis and periodontitis, depending on disease severity including gingival irrigations, mechanical instrumentation, local and systemic antimicrobial therapy, and surgical treatment [11,12].

Oxidative stress productions from different biological sources are due to an imbalance of natural antioxidants, which further leads to various inflammatory-associated diseases [13]. Antioxidant activity depends on the structure of functional groups. The number of hydroxyl groups greatly influences several mechanisms of antioxidant activity such as scavenging radicals and metal ion chelation ability [14].

Numerous plants have historically been considered to be effective in preventing inflammation [15]. *Allium sativum (Liliaceae)*, *Bauhinia variegata* (Fabaceae), *Eupatorium perfoliatum* (Asteraceae), and *Kalopanax pictus* inhibited inflammation by reducing the levels of IL-6, TNF, interferon, nitric oxide (NO), and Prostaglandin E_2_ (PGE2) of lipopolysaccharide (LPS)-stimulated macrophages and peripheral blood mononuclear cells (PBMCs) [16,17,18,19]. In an in vivo study, extract of *Nigella sativa* and *Geranium nepalense* exhibited significant anti-inflammatory activity on carrageenan-induced paw edema and tetradecanoyl phorbol acetate (TPA)-induced mouse ear edema [20,21]. Another study showed anti-inflammatory effect of protein fraction of *Calotropis procera* latex by inhibiting the expression of iNOS, COX-2, TNF-α, and IL-1β on 5-fluorouracil induced oral mucositis in golden hamsters [22].

*Moringa oleifera* Lam. (MO) provides beneficial nutrients and medical properties. MO is known as the miracle tree, with various therapeutic properties such as hepatoprotective, antioxidant, anti-inflammatory, anti-ulcer, anti-cancer, anti-hypoglycemic, anti-plasmodic, anti-bacterial, and anti-fungal activities [23,24]. Furthermore, MO leaves present a high content of vitamins and antioxidant compounds such as vitamin A, vitamin C, vitamin E, carotenoids, polyphenols, flavonoids, phenolic acids, alkaloids, tannins, and saponins. MO leaves have strong antioxidant activity, mainly due to their high content of bioactive polyphenols [25,26]. Many research studies have been conducted on medicinal plants and their anti-inflammatory effects on the expression of pro-inflammatory mediators, including NO, nitric oxide synthase (iNOS), COX-2, IL-1β, IL-6, and TNF-α. Certain plants have been shown to increase the level of anti-inflammatory cytokine IL-10 [27,28,29]. Our previous study found that bioactive compounds in MO leaf extract reduced the production of pro-inflammatory cytokines, including TNF, IL-6, IL-8, and COX-2 of LPS-induced human monocyte-derived macrophages (MDMs) via inactivation of NF-κB, blocking both IκB-α degradation and nuclear translocation of NF-κB (p65) [30,31]. In addition, we also found that pre-treating human T-cells with MO leaves extract inhibited reduction in IL-2 production, down-regulation of *IL2* gene expression, and DNA damage in T-cells treated with oxidative substances [32]. *M. oleifera* leaf fraction was reported to have anti-inflammatory properties by inhibiting LPS-induced production of NO and pro-inflammatory cytokines in RAW264.7 cells [33]. Another study identified that bioactive isothiocyanates from MO leaf extract significantly inhibited the expression of iNOS, IL-1β, and the production of NO and TNF-β [34].

Astragalin (kaempferol-3-O-β-D-glucoside) is a bioactive natural flavonoid. This compound exhibits anti-inflammation through NF-κB signaling pathway in macrophage and mice model [35,36]. Astragalin also has antioxidant property [37]. This compound has been used as a chemical marker for quality control and standardization of MO extract using the high performance liquid chromatography (HPLC) method [38].

*Cyanthillium cinereum* (Less.) H. Rob. (CC) has various medicinal properties such as analgesic, anti-pyretic [39], anti-bacterial [40], and anti-fungal activities [41]. In addition, CC decreases cigarette craving in smokers [42,43] and provides anti-inflammation and anti-microbial biological activity [44]. *C. cinereum* contains antioxidant compounds such as tannins, catechins, and flavonoids that exhibited protective activity of 2,2′-azobis(2-amidinopropane) dihydrochloride (AAPH) oxidation in human red blood cells in an ex vivo model [45].

*M. oleifera* leaf and over ground parts of CC extracts provide anti-inflammatory activity that might reduce gingivitis and oral inflammation in smokers. Thus, in the present study, candy lozenges containing major active ingredients of MO leaf and over ground parts of CC extracts were investigated for anti-oral inflammation in volunteer smokers. We hypothesized that MO leaf, CC extract and their products contain high contents of phenolic compounds and antioxidant potential. Moreover, MO lozenges and MO + CC lozenges might reduce gingivitis and oral inflammation in smokers.

## 2. Results

### 2.1. Quality Control of Lozenges

**Figure 1:**
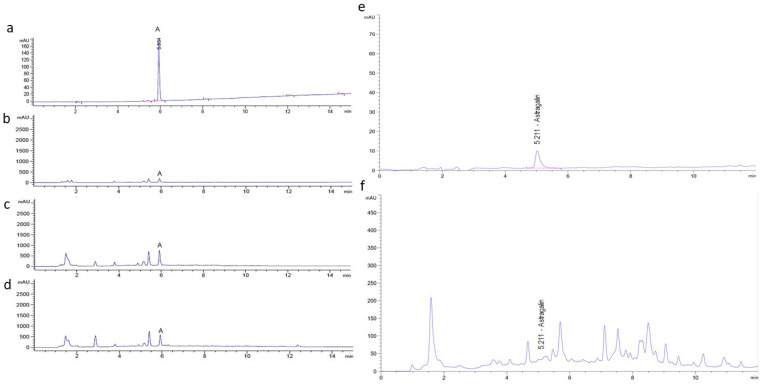
HPLC system for determination of astragalin at 267 nm. Typical HPLC chromatograms of (**a**) astragalin standard at 20 µg/mL, (**b**) MO leaf extract (3 mg/mL), (**c**) MO lozenges, (**d**) MO + CC lozenges, (**e**,**f**) astragalin standard and CC extract (3 mg/mL), respectively.

Quality control of lozenges including stability, contamination testing (impurity, microbial, metal), and moisture analysis was evaluated by Khaolaor Laboratories Co., Ltd. Contaminations of lead (Pb), arsenic (As), and cadmium (Cd) were not found in all samples. Contamination of yeast and mold < 10 colony-forming unit (cfu)/g was found in all types of lozenges. Bacteria including Salmonella spp., Clostridium spp., and Staphylococcus aureus were not found (data not shown). Residual solvent of ethanol was measured using GC/MS with headspace technique by Central Laboratory (Thailand) Co., Ltd. Residual ethanol solvent in the MO and CC extracts were 57,400 ppm and 28,800 ppm, respectively. However, residual solvent in the placebo, MO lozenges, and MO + CC lozenges was not detected (Table 1). Content of astragalin in MO, CC extract, and lozenges was measured by high performance liquid chromatography (HPLC). Typical chromatograms of the astragalin standard are shown in Figure 1a–f. The retention time (RT) of astragalin (20 µg/mL) was 5.934 min, while astragalin in MO leaf extract, MO lozenges, and MO + CC lozenges had the same RT (Figure 1a–d). Astragalin in CC extract was eluted at 5.211 min, similar to the astragalin standard (Figure 1e,f). Average astragalin contents in MO and CC extracts were 5914.70 ± 260.36 µg/g extract and 245.72 ± 8.81 µg/g extract, respectively, while astragalin contents of 215.87 ± 19.57 µg/tablet and 176.69 ± 21.17 µg/tablet were found in MO lozenges and MO + CC lozenges, respectively (Table 2).

### 2.2. Total Phenolic Content

**Figure 2:**
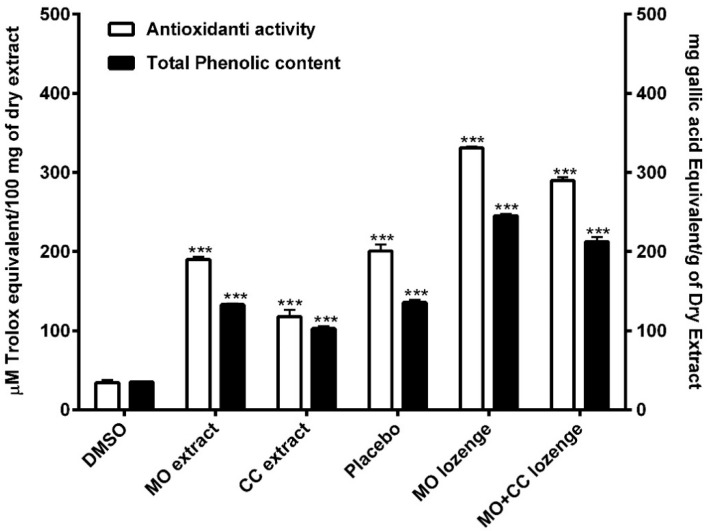
Antioxidant activity of extracts and lozenges are shown in white bars. Total phenolic contents of extracts and all lozenges are presented in black bars. DMSO: dimethyl sulfoxide; MO extract: *Moringa oleifera* extract; CC extract: Cyanthillium cinereum extract; MO lozenges: Moringa lozenges; MO + CC: Moringa and Cyanthillium lozenges. Data are presented as means ± SEM. *** *p* < 0.001, compared to DMSO.

The total phenolic contents in MO and CC extracts were 132.98 ± 2.14 and 102.81 ± 4.07 mg gallic acid equivalent (GAE)/g of dry extract, respectively. The phenolic contents in placebo, MO lozenges and MO + CC lozenges were 135.71 ± 5.34, 245.14 ± 3.8, and 212.42 ± 9.82 mg GAE/g of dry extract, respectively. Total phenolic content of MO extracts and all lozenges were significantly higher compared to DMSO (34.23 ± 1.53 mg GAE/g of dry extract) (Figure 2). These results indicated that MO extracts, CC extracts, and all lozenges contained high amounts of phenolic compounds.

### 2.3. Anti-Oxidant Activity

The free radical scavenging effects of plant extracts and all lozenges were significantly higher compared to DMSO (34.65 ± 5.04 µM Trolox equivalent (TE) /100 mg of dry extract). MO leaf extract and CC extract showed antioxidant activity at 189.93 ± 5.24 and 117.71 ± 15.02 µM TE/100 mg of dry extract, respectively. The anti-free radical activity of MO lozenges was highest at 330.79 ± 3.13 µM TE/100 mg of dry extract, while the antioxidant of placebo and MO + CC lozenges were 200.56 ± 14.44 and 289.79 ± 7.06 µM TE/100 mg of dry extract, respectively (Figure 2). These findings demonstrated that MO extracts, CC extracts, and all lozenges provided high antioxidant potential.

### 2.4. Subjects Characteristic and Clinical Laboratory Measurement

At week 4, 25 subjects were loss to follow-up, and 67 subjects had completely done the process. The mean ± SD age of smokers in placebo, MO Lozenge, and MO + CC lozenge group were 37.14 ± 10.06, 39.77 ± 11.93, and 29.13 ± 10.25, respectively. The placebo group had mean ± SD of body mass index (BMI) of 21.68 ± 4.6 while MO Lozenge, and MO + CC lozenge group had BMI of 20.72 ± 3.59 and 23.7 ± 4.05, respectively. There was not significant difference in age and BMI between groups (Table 3). Blood pressure (BP), heart rate (HR), fasting blood sugar (FBS), aspartate aminotransferase (AST), alanine aminotransferase (ALT), alkaline phosphatase (ALP), blood urea nitrogen (BUN), and serum creatinine (sCr) in each group at the end of using lozenge were not significantly different from baseline visit. The summarized of clinical laboratory characteristics of smokers was shown in Table 4.

### 2.5. The Anti-Inflammation of Lozenge in Smokers

To investigate whether lozenge had an anti-oral inflammatory effect in smokers, all subjects were received three lozenges per day. After 4 weeks, the change of oral inflammation and gingival condition of subject were done by oral examination and gingival index (GI) by the dentist. The mean ± SD of oral inflammation between baseline and week 4 in all groups were not significantly decrease. There was not significant difference of oral inflammation in between groups as well. However, percentage decrease of oral inflammation in group II (MO lozenge) were significantly higher compared to placebo group. Interestingly, the GI from baseline to week 4 in group III (MO + CC lozenge) was significantly decreased. However, the GI was not significantly difference in group I (Placebo) and group II (MO lozenge). Moreover, percentage decrease of GI in group II (MO lozenge) and group III (MO + CC lozenge) were significantly higher compared to placebo group. This result confirmed that MO and MO + CC lozenge showed inhibitory effect of gingivitis and oral inflammation in smokers. The change of GI and oral inflammation in each group as shown in Table 5.

### 2.6. The Satisfaction and Lozenge Usage of Smokers and Control Group

Percentage of lozenge using (mean ± SD) in group I (Placebo), group II (MO lozenge), and group III (MO + CC lozenge) were 83.64 ± 22.08, 84.33 ± 20.26, and 78.19 ± 25.99, respectively. The score of satisfaction on product about lozenge appearance of placebo, MO lozenge, and MO + CC lozenge were 3.91 ± 0.84, 3.76 ± 0.83, and 3.83 ± 0.83 while score of lozenges flavor were 3.86 ± 0.89, 3.57 ± 0.87, and 3.43 ± 1.24, respectively. However, there was not significantly difference between groups (Table 6).

## 3. Discussion

This was a double-blinded randomized controlled clinical trial. Herbal lozenges consisting of *M. oleifera* leaf extract and over ground parts of *C. cinereum* extract were developed for investigation of oral inflammation and gingivitis in volunteer smokers at the smoking cessation clinic, Wang Thong Hospital, Phitsanulok, Thailand.

Both plants, MO and CC have historically been used in traditional medicine, MO leaf showed high pharmacological properties including hepatoprotective, antioxidant, anti-inflammatory, anti-ulcer, anti-cancer, anti-bacterial, anti-fungal, and anti-diabetes [23,24]. *C. cinereum* provides various medicinal potential such as analgesic, antioxidant, anti-inflammation, anti-pyretic, antibacterial, and anti-fungal activities [46,47]. Both plants had strong anti-inflammatory properties. A previous study reported that CC extract significantly inhibited the secretion of IFN-γ in a dose-dependent fashion, while IL-10 anti-inflammatory cytokine increased [48]. Identified isothiocyanates from MO leaf extract significantly decreased gene expression and production of inflammatory mediators in RAW macrophages [34]. Moreover, the effects of different solvent fractions including butanol, ethyl acetate, chloroform, and hexane of MO extract decreased IL-6, TNF-α, IL-1β, and prostaglandin E2 (PGE2) production in LPS-stimulated RAW264.7 macrophages after treatment with each extracted solvent [33]. Our previous study found that MO leaf extract and contained bio-active compounds reduced the production of pro-inflammatory cytokines including IL-6, IL-8, and TNF by suppressing phosphorylation of IκB-α and nuclear translocation of NF-κB (p65) [30,31].

Both MO and CC plants are rich sources of antioxidant compounds that provide antioxidant activity [26,45]. Our results showed MO leaf extract, CC extract, MO lozenges and MO + CC lozenges contained high total phenolic contents with greater DPPH radical scavenging activity compared to the control as DMSO (*p* < 0.001). These finding supported previous studies from Malaysia and Nigeria where fractions and crude extracts of CC demonstrated antioxidant activity that related to the amount of total phenolic and total flavonoid contents in the plant [49,50]. Leaf of MO also showed high potential as a natural source of antioxidants by displaying highest DPPH and ABTS radical scavenging and FRAP total reducing power activities [51,52,53]. Imbalance between antioxidants and free radicals can lead to oxidative damage of cellular molecules such as DNA, proteins, and lipids. Oxidative stress activates a variety of inflammatory mediators involved in several chronic diseases [14]. Our findings suggested that MO leaf extract, CC extract, MO lozenges, and MO +CC lozenges had high amounts of phenolic compounds with strong antioxidant activity. These plants showed promise for the development of further antioxidant and anti-inflammatory therapeutic products.

Astragalin, a bioactive natural flavonoid has been well known for multiple pharmacological properties including antioxidant, anti-inflammation, anti-cancer, and cardioprotective property [37]. Furthermore, astragalin has been used as a chemical marker for quality control and standardization of MO extract using the high performance liquid chromatography (HPLC) method [38]. The quality control of plant extraction and its products can be done by checked the present of an active ingredient. Hence, we investigated astragalin in our plant extracts and lozenges using HPLC. The resulted revealed that MO and CC extracts as well as MO and MO + CC lozenges contained bioactive marker, astragalin. This indicated that plant extracts were potential active ingredient in lozenges.

Smoking is one of the risk factors for oral inflammation and periodontitis with high amount of plaque accumulation [5]. Dental plaque biofilm has biologically active products as Gram-positive and Gram-negative bacteria that colonize the tooth surface around the gingival border and interproximal areas. These products include endotoxins, cytokines, and protein toxins that penetrate the gingival epithelium and result in gingivitis [54]. Thus, oral hygiene and control of bacterial plaque biofilm formation are effective methods to prevent gingivitis [10]. *M. oleifera* leaf showed medicinal properties that improved human oral health care. MO leaf extract inhibited cariogenic biofilm formation and acted against oral pathogens including *Staphylococcus aureus*, *Streptococcus mutans* and *Candida albicans* [55,56,57], whereas CC showed significant results for anti-inflammatory and antimicrobial action on *S. aureus* [44,58]. This present study revealed that the GI from baseline to week 4 in smoker group III (MO + CC lozenges) significantly decreased, indicating that MO + CC lozenges reduced gum inflammation in smokers.

This was supported by a previous report from Egypt where MO leaf extract significantly decreased periodontal inflammation by reducing leptin and IL-6 serum levels in a male albino rat periodontal model [59], while MO leaf extract also reduced production of TNF-α and IL-1β in the gum tissue in a male Wistar rat periodontal model (*p* ≤ 0.001) [60]. Moreover, a variety of products from MO leaf are available for cleaning the teeth and gums, and treatment of oral ailments such as MO tooth powder, MO solution, mouthwash, and toothpaste [61]. However, this present study, the percentage decrease of oral inflammation in the placebo group was possibly caused from the activity of peppermint essential oil and menthol essential oil, which displayed antioxidant and anti-inflammation activities [62,63]. Essential oil also reduced gingival inflammation in orthodontic patients [64].

Furthermore, CC decreased cigarette by their active compounds including flavonoids and hirsutinolides can inhibit cytochrome P450 2A6 (CYP2A6), monoamine oxidase (MAO)-A, and MAO-B, which reduced nicotine and dopamine metabolism [46]. Previous clinical study evaluated the effect of CC infusion tea bag in sixty-four subjects at outpatient smoking cessation clinic at Thanyarak Institute, Thailand. They found that the 7-day point prevalence abstinence rates (PAR) and continuous abstinence rates (CAR) were increased in CC group compared to the placebo [42]. Our present study showed the percentage decrease of oral inflammation in group II and GI in both groups II and III were significantly higher than in the placebo group (*p* ≤ 0.001). These results suggested that MO lozenges and MO + CC lozenges provided anti-oral inflammation as a protective effect on gingivitis in smokers and might be use as alternative treatment for smoking cessation.

## 4. Materials and Methods

### 4.1. Chemicals and Reagents

Methanol and 95% ethanol were provided from RCI Labscan Limited, Thailand. Astragalin and trolox were supplied from Sigma-Aldrich, St. Louis, MO, USA. Folin-Ciocalteu reagent, garlic acid as well as DPPH reagent were purchased from EMD Millipore Corp., Billerica, MA, USA.

### 4.2. Plant Extraction

*M. oleifera* Lam. (MO) leaf extract (Lot. No. RD190514) and *C. cinereum* (Less.) H. Rob. (CC) extract (Lot. No. RD190515) were obtained from Khaolaor Company (Samut Prakan, Thailand). Briefly, dried MO leaf powder (8 kg) and over ground parts of CC powder (4 kg) were successively extracted twice with 95% ethanol (24 and 12 L) at room temperature for 7 days. Extracts were filtered using Whatman Qualitative Filter Paper No.1 with pore size 11 µm and the solvent was removed by a rotary evaporator. After the evaporation of solvents, 854 g of crude MO leaf extract (10.68% yield) and 172 g of CC extract (4.3% yield) were obtained. This method was modified from a previous study [65].

### 4.3. Dose Calculation of MO and CC Extract in Lozenge

Concentration of MO and CC extracts in lozenges were calculated using no observed adverse effect level determination (NOEL) and human equivalent dose (HED) as previous described [66]. Previous study of NOEL of MO and CC extract were 450 and 400 mg/kg in rat [67]. Thus, HED of MO and CC extract were 72.93 and 64.82 mg/kg. The maximum recommended starting dose (MRSD) for clinical trial was determined and 60 kg using as average weight of volunteers. The safety concentrations of MO leaf and CC extract per day were 437.58 and 388.92 mg/day, respectively. In this clinical trial, volunteers were received 3 lozenges per day, dose of MO leaf and CC extract per lozenge were 145.86 mg/lozenge and 129.64 mg/lozenge, respectively.

### 4.4. Lozenge Formulation and Evaluation

In this study, three hard lozenges were developed as the placebo, Moringa (MO) lozenge, and Moringa and Cyanthillium (MO + CC) lozenge. The hard candy lozenges consisted of sucralose, glucose syrup, peppermint essential oil, menthol essential oil, citric acid and active ingredients including MO and CC extract. Concentration of MO and CC extract in lozenges were calculated using NOEL and HED as previous described [66]. Hard candy lozenges were made by dissolving sucralose and syrup in water to prepare a candy base. The candy was heated until the temperature reached 145–156 °C, then cooled followed by addition of acidulants and flavoring agents. Active ingredients including MO and CC extracts were added. The candy mass was transferred to candy molds. The lozenge mass was checked, and the obtained lozenges were packaged in single unit wrappers [68]. Large batches of lozenges were produced, with quality control evaluated by Khaolaor Laboratories Co., Ltd. for stability, contamination testing (impurity, microbial, metal), and moisture analysis. Residual solvent of ethanol in extract was measured using GC/MS with headspace technique by Central Laboratory (Thailand) Co., Ltd. Astragalin was used as the chemical marker of MO and CC extracts. Astragalin is also known to have antioxidant properties [38]. Hence, astragalin was used as a bioactive marker for quality control and standardization of extract and lozenge products. Contents of astragalin in extracts and lozenges were measured by high performance liquid chromatography (HPLC).

### 4.5. Gas Chromatography Mass Spectroscopy (GC/MS) Analysis

The residual solvent in sample was measured by G1888 HS sampler with a 6890N series GC equipped with a Dean’s Switch, FID, and 5973 series MS (Agilent Technologies, Palo Alto, CA, USA). The analytical column was DB-ALC1 (Agilent Technologies, Palo Alto, CA, USA) fused-silica capillary column with dimensions of 30 m × 0.25 mm i.d. and 0.25 µm film thickness. The Dean’s Switch was configured using a 1:1 split ratio to the FID and MS. Helium was used as the carrier gas. The HS loop and transfer line temperatures were set at 70 and 90 °C, respectively. The vial pressurization was set at 15 psi for 0.15 min. Injection, loop fill, and loop equilibration times were set at 0.50, 0.15, and 0.05 min, respectively. The GC cycle time was set at 13.5 min. For the GC, a constant helium flow rate of 3 mL/min was used. The injection port temperature was maintained at 90 °C with a 5:1 split injection of the headspace and a septum purge flow of 3 mL/min. The initial GC oven temperature of 35 °C was held for 3 min and then ramped at 25 °C/min to final temperature of 90 °C, which was held for 4.3 min. The total GC run time was 8.5 min/sample. Both restrictors were set at constant helium flow of 2 mL/min. The FID temperature was maintained at 300 °C with hydrogen. Air and constant column plus helium makeup pressures of 40, 450, and 50 psi, respectively. The MS transfer line was maintained at 280 °C and the scan range was set from 20 to 200.

### 4.6. High Performance Liquid Chromatography (HPLC) Analysis

Astragalin contents in MO extract, MO lozenges, and MO + CC lozenges were measured using HPLC (Agilent Technologies 1260 Infinity-DAD Detector). Standard stock solution of astragalin (97% purity, HPLC grade from Sigma-Aldrich, St. Louis, MO, USA) was prepared by dissolving the substances in 20% acetonitrile to a final concentration of 1940 µg/mL. Standard working solutions were prepared by diluting the stock solutions at 0.3125–100 µg/mL. The calibration curve of astragalin was successfully constructed using the peak area of the standard (y axis) and known concentration of the standard (μg/mL; x axis). The extract was dissolved in 20% acetonitrile at 3 mg/mL before injection to HPLC. For sample preparation, three tablets of each lozenge type were extracted using 3 mL of 20% acetonitrile and sonicated for 20 min. The sample was further centrifuged at 6000 rpm for 5 min and the supernatant was filtered using a 0.45 µm nylon syringe filter. The injection volume of all samples was 20 µL. The HPLC analysis was performed using a Phenomenex Luna C-18(2), 150 × 4.6 mm, 5 µm column. The mobile phase was gradient elution of 15–85% acetonitrile in water for 15 min, and post-run was set for 3 min with a flow rate of 1 mL/min. While CC extract was measured using HPLC Agilent Technologies 1290 Infinity-DAD Detector. The HPLC analysis was performed using C-18, 2.1 × 100 mm, 1.8 µm column. The mobile phase was gradient elution of acetonitrile in water with a flow rate of 0.15 mL/min. The analysis of all samples was monitored at 267 nm.

### 4.7. Determination of Total Phenolic Contents

The phenolic contents of MO, CC extracts, MO lozenges and MO + CC lozenges were determined using the Folin-Ciocalteu method. Dry samples were dissolved in 50% methanol to reach a final concentration of 100 mg/mL. Samples were diluted in 100 µL of deionized water and mixed with 5 µL of Folin–Ciocalteu reagent (EMD Millipore Corp., Billerica, MA, USA). After 5 min incubation at room temperature, 100 µL of 2% sodium carbonate (Na_2_CO_3_) was added into a 96 well plate. The reaction mixtures were incubated at 50 °C for 60 min for the development of color, and the absorbance was measured at 750 nm using an EnSpire^®^ Multimode microplate reader (PerkinElmer, Inc., Waltham, MA, USA). Gallic acid (EMD Millipore Corp., Billerica, MA, USA) was used as a standard curve, and total phenolic contents were expressed in milligram gallic acid equivalent (GAE) per gram of dry extract. This method was modified from a previous study [69].

### 4.8. Determination of Antioxidant Activity

DPPH (2,2-diphenyl-1-picryl-hydrazyl-hydrate) radical scavenging assay was used to evaluate the antioxidant activity of MO, CC extracts, MO lozenges and MO + CC lozenges. This assay was modified from a previous method [70]. A preparation of 0.1 mM DPPH solution was dissolved in 0.6 mM of stock solution with methanol. The extracts were dissolved in dimethyl sulfoxide (DMSO) to a final concentration of 100 mg/mL and was added with 5 µL of extract in a 96 well plate. Then 50 µL of DPPH reagent (EMD Millipore Corp., Billerica, MA, USA) was added and the plate was incubated at room temperature for 15 min, after which the absorbance of the solution was measured at 540 nm using an EnSpire^®^ Multimode microplate reader (PerkinElmer, Inc., Waltham, MA, USA). A standard curve was obtained by Trolox (Sigma-Aldrich, St. Louis, MO, USA). The antioxidant activity of the MO extract was calculated as Trolox equivalent.

### 4.9. Study Design

**Figure 3:**
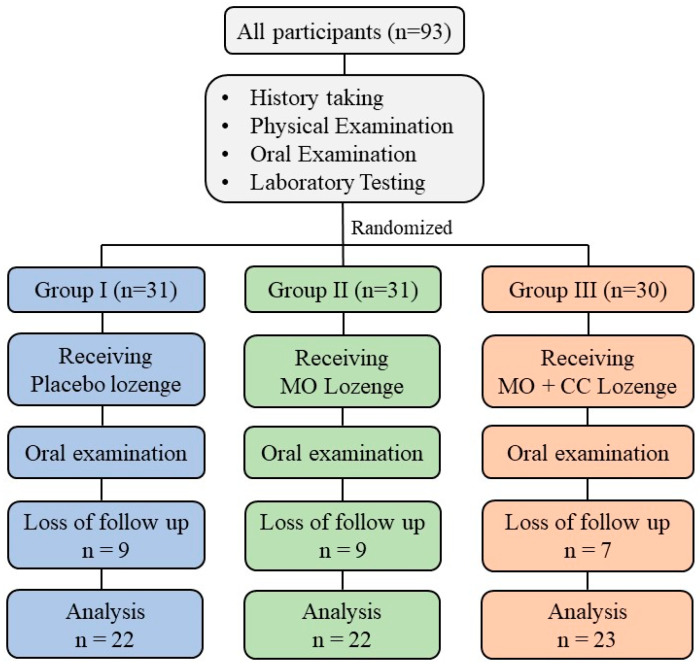
Study design.

This study, a double-blinded randomized controlled clinical trial was conducted at the smoking cessation clinic, Wang Thong Hospital, Phitsanulok, Thailand. The study protocol was approved by the Human Ethics Committee of Naresuan University (IRB no. 0346/62). All subject who met the inclusion criteria were included in this study and the enrolled volunteers were written inform consent with the Declaration of Helsinki. To test efficacy of lozenges, 93 smokers aged 18–65 years were selected and randomly divided into 3 groups. The subjects in each group were assigned to get the different type of lozenges. Group I received placebo, Group II and group III received MO lozenge and MO + CC lozenge, respectively. The design of study procedure is shown in Figure 3.

### 4.10. Subjects

Smokers were at least 18 years to 65 years of age from smoking cessation clinic, Wang Thong Hospital, Phitsanulok, Thailand, smoked more than 5 cigarettes/day in the past 6 months and able to communicate in Thai fluently. Smokers had no period of abstinence more than 3 months in the past year and never participate in other smoking cessation clinic at least 2 weeks. Inclusion criteria were including (i) smokers ages between 18 and 65 years; (ii) smoked more than 5 cigarettes/day in the past 6 months; (iii) subjects have been diagnosed by a dentist with mild or moderated of oral inflammation; and (iv) healthy subjects with no history of allergic to any ingredient in lozenges.

The exclusion criteria were (i) subjects had a history of disease including diabetes mellitus, cardiovascular, cancer, significant hepatic and renal impairment, neurologic and psychiatric disorder, and immunodeficiency; (ii) pregnant or breastfeeding; (iii) subjects who use drugs including antibiotics, anti-inflammatory, anticoagulants, anticonvulsants, immunosuppressive drugs within 6 months prior to the study visit; (iv) severe alcoholism or drug addict; and (v) previous use of anti-inflammation or smoking cessation products within a month.

Discontinuation criteria were (i) participants experienced an adverse effect such as severe allergic of gum/oral cavity, abnormal of gastrointestinal tract; (ii) researchers are unable to contact volunteers; and (iii) volunteers do not cooperate with research agreement.

### 4.11. Sample Size

Sample size for two independent samples was calculated using the formula modified from a previous study [71] as follows:

$$\begin{array}{l} n=\frac{(Z\alpha +Z\beta {)}^{2}\times 2P^{\prime }\left(1-P^{\prime }\right)}{D^{2}}\\ \;=\frac{{\left(1.96+0.84\right)}^{2}\times 2\left(0.5\right)\left(1-0.5\right)}{{\left(0.69-0.31\right)}^{2}}\\ \;=27.14 \end{array}$$

where, P′= P1 + P2/2 and D = P1 − P2

n = sample size
Zα = The critical value from the standard normal distribution, α = 0.05 and Zα = 1.96.
Zβ = The critical value from the standard normal distribution, β = 0.2 and Zβ= 0.84
P1 = Mean of population 1 or study group
P2 = Mean of population 2 or control group
*D* = Expected mean difference between the study group and the control group

Sample size was 27 subjects per group, with follow-up period of 4 weeks and expected dropout rate of 15%. Therefore, the number of subjects in each group should be 31.

### 4.12. Study Procedure

On day 0, selected smokers following the above criteria signed an informed consent form. Physical examination including body weight and blood pressure were recorded. A blood sample (5 mL) was drawn for laboratory testing including fasting blood sugar (FBS), aspartate aminotransferase (AST), alanine aminotransferase (ALT), alkaline phosphatase (ALP), blood urea nitrogen (BUN), and serum creatinine (sCr). Oral inflammation and gingival index (GI) of individual subjects were measured by a dentist.

The oral examination was evaluated with 6 scores including 0 = absence of oral inflammation, 1 = very mild oral inflammation, 2 = mild oral inflammation, 3 = moderate oral inflammation, 4 = quite severe oral inflammation, and 5 = severe oral inflammation. This oral scoring system was followed previous clinical trial research [72]. GI was evaluated using modified gingival index (MGI) with 5 rating score between 0 and 4, including 0 = normal gingiva, 1 = mild inflammation or with slight changes in color and texture but not in all portions of gingival marginal or papillary, 2 = mild inflammation, slight changes in color and texture in all portions of gingival marginal or papillary, 3 = moderate, bright surface inflammation, erythema, edema and/or hypertrophy of gingival marginal or papillary, and 4 = severe inflammation: erythema, edema and/or marginal gingival hypertrophy of the unit or spontaneous bleeding, papillary, congestion or ulceration. This method was previously described [73].

All subjects were randomly divided into three groups: group I (placebo), group II (MO lozenges), and group III (MO + CC lozenges). The subjects were given a pack of lozenges for the 4 weeks of the study period, and all subjects were blinded to treatment assignments. They were asked to take three lozenges daily, with one lozenge after breakfast, lunch, and dinner. The follow-up period was 4 weeks with two clinic visits (weeks 0 and 4) and four telephone contacts (weeks 1–4). At the end of the 4-week study, all subjects were asked to attend the smoking cessation clinic for history taking, physical examination, oral examination, and blood testing of FBS, AST, ALT, ALP, BUN, and sCr. After using the lozenges, subjects also evaluated product satisfaction using a 5-point hedonic scale questionnaire including 1 = like very slightly, 2 = like slightly, 3 = like moderately, 4 = like very much, and 5 = like extremely.

### 4.13. Statistical Analysis

One-way ANOVA and Kruskal–Wallis test were used for data analysis among three groups. The paired T-test and Wilcoxon signed rank test were used to compare the difference of data between baseline and week 4. GraphPad Prism software and SPSS were used to analyze all parameters in this study. A confidence interval of 95% (*p* = 0.05) was used in all statistical analyses.

## 5. Conclusions

This study revealed that *M. oleifera* and *C. cinereum* extract showed high potential of antioxidant. Findings suggested that lozenges containing Moringa and a combination of Moringa and Cyanthillium extracts had strong pharmacological effect for reducing gingivitis and oral inflammation in healthy smokers. This in vivo study indicated that Moringa and Cyanthillium medicinal plant extracts are safe for humans and could be used to improve oral health care, especially in smokers with high prevalence of gingivitis and periodontitis.

## Figures and Tables

**Table 1:** Residual solvent.

Sample	Residual Solvent	
	% (*w*/*v*)	ppm
MO leaf extract	5.74	57,400
CC extract	2.88	28,800
Placebo	Not detected	-
MO lozenge	Not detected	-
MO + CC lozenge	Not detected	-

**Table 2:** Concentration of astragalin in sample. The values are the mean ± SD (*n* = 3). MO: *Moringa oleifera*; CC: *Cyanthillium cinereum* (Less.) H. Rob.

Sample	Astragalin Content
MO leaf extract (µg/g extract)	5914.70 ± 260.36
CC extract (µg/g extract)	245.72 ± 8.81
MO lozenge (µg/tablet)	215.87 ± 19.57
MO + CC lozenge (µg/tablet)	176.69 ± 21.17

**Table 3:** The characteristics data of subjects in placebo, MO lozenge, and MO + CC lozenges groups. The different of age and body mass index (BMI) among three groups were analyzed by one-way ANOVA at the *p* < 0.05 level. The values are the mean ± SD, ns is not significant. MO: *Moringa oleifera*; CC: *Cyanthillium cinereum*.

	Placebo	MO Lozenge	MO + CC Lozenge	*p*-Value
	(*n* = 22)	(*n* = 22)	(*n* = 24)	
Sex: Male	22	22	23	
Age (years)	37.14 ± 10.06	39.77 ± 11.93	29.13 ± 10.25	ns
BMI (kg/m^2^)	21.68 ± 4.6	20.72 ± 3.59	23.48 ± 4.05	ns

**Table 4:** Laboratory examination of smokers at baseline and week 4. The different of blood pressure (BP) and heart rate (HR) were analyzed using paired T-test (*p* < 0.05). The different of fasting blood sugar (FBS), aspartate aminotransferase (AST), alanine aminotransferase (ALT), alkaline phosphatase (ALP), blood urea nitrogen (BUN), and serum creatinine (sCr) were analyzed by Wilcoxon signed rank test at the *p* < 0.05 level. The values are the mean ± SD and ns is not significant. MO: *Moringa oleifera*; CC: *Cyanthillium cinereum*.

Parameters		Placebo (*n* = 22)		*p*-Value	MO Lozenge (*n* = 22)		*p*-Value	MO + CC Lozenge (*n* = 23)		*p*-Value
		Baseline	Week 4		Baseline	Week 4		Baseline	Week 4	
BP (mmHg)	Systolic	132.25 ± 15.3	124.37 ± 11.46	ns	134 ± 14.92	139.63 ± 12.73	ns	133.81 ± 12.04	131.73 ± 17.41	ns
	Diastolic	90.75 ± 14.91	82.12 ± 9.73	ns	87.63 ± 11.55	92.63 ± 10.08	ns	88.06 ± 12.28	79.6 ± 23.1	ns
HR		84.87 ± 18.19	88 ± 12.99	ns	90.72 ± 18.34	95.36 ± 17.23	ns	95.81 ± 18.41	92.06 ± 21.11	ns
FBS (mg/dL)		89 ± 11.95	92.62 ± 25.11	ns	96.54 ± 21.69	103.54 ± 19.30	ns	97.93 ± 9.65	99.38 ± 15.24	ns
AST (U/L)		58 ± 85.78	29.62 ± 11.78	ns	66.18 ± 83.59	55.45 ± 70.01	ns	36.81 ± 30.03	29.87 ± 10.36	ns
ALT (U/L)		30.12 ± 25.44	24.87 ± 14.97	ns	48.27 ± 50.88	38.09 ± 32.34	ns	36.75 ± 41	25.68 ± 30.26	ns
ALP (U/L)		123.75 ± 73.24	104.75 ± 67.03	ns	99 ± 59.15	106.48.10	ns	86.81 ± 42.95	95.18 ± 62.25	ns
BUN (mg/dL)		11.97 ± 3.46	11.98 ± 2.98	ns	12.79 ± 2.69	11.94 ± 3.23	ns	12.35 ± 3.39	13.81 ± 3.28	ns
sCr (mg/dL)		0.86 ± 0.15	0.91 ± 0.14	ns	0.98 ± 0.19	1 ± 0.2	ns	0.98 ± 0.12	0.99 ± 0.12	ns

**Table 5:** Gingival index and oral inflammation smokers in placebo, MO lozenge, and MO + CC lozenge groups. The different of oral inflammation and gingival index among three groups were analyzed using Kruskal–Wallis test. The values between baseline and week 4 was analyzed by Wilcoxon signed rank test at the *p* < 0.05 level. The values were represented as mean ± SD, ns is not significant. MO: *Moringa oleifera*; CC: *Cyanthillium cinereum*.

Parameters	Placebo	MO Lozenge	MO + CC Lozenge	*p*-Value
	(*n* = 22)	(*n* = 22)	(*n* = 24)	
Oral Inflammation				
Baseline	0.14 ± 0.35	0.27± 0.55	0.21 ± 0.25	ns
Week 4	0.05 ± 0.21	0.05 ± 0.22	0.09 ± 0.42	ns
*p*-Value	ns	ns	ns	
% Decrease	64.28	81.48	57.14	<0.001
Gingival Index				
Baseline	1.77 ± 0.69	2.18 ± 1.59	1.88 ± 0.61	ns
Week 4	1.55 ± 0.51	1.62 ± 0.59	1.48 ± 0.51	ns
*p*-Value	ns	ns	<0.05	
% Decrease	12.43	25.69	21.28	<0.001

**Table 6:** The satisfaction and lozenge usage after 4 weeks. The different of lozenge usage and the satisfaction among three groups were analyzed using Kruskal–Wallis test at *p* < 0.05. The values were represented as mean ± SD, ns is not significant. MO: *Moringa oleifera*; CC: *Cyanthillium cinereum*.

Parameters	Placebo	MO Lozenge	MO + CC Lozenge	*p*-Value
	(*n* = 22)	(*n* = 22)	(*n* = 24)	
Lozenge Usage (%)	83.64 ± 22.08	84.33 ± 20.26	78.19 ± 25.99	ns
Lozenge Appearance	3.91 ± 0.84	3.76 ± 0.83	3.83 ± 0.83	ns
Flavor	3.86 ± 0.89	3.57 ± 0.87	3.43 ± 1.24	ns

## References

[1] Cunningham F., Fiebelkorn S., Johnson M., Meredith C. (2011). A novel application of the Margin of Exposure approach: Segregation of tobacco smoke toxicants. Food Chem. Toxicol..

[2] Lee J., Taneja V., Vassallo R. (2012). Cigarette smoking and inflammation: Cellular and molecular mechanisms. J. Dent. Res..

[3] Arnson Y., Shoenfeld Y., Amital H. (2010). Effects of tobacco smoke on immunity, inflammation and autoimmunity. J. Autoimmun..

[4] Rom O., Avezov K., Aizenbud D., Reznick A.Z. (2013). Cigarette smoking and inflammation revisited. Respir. Physiol. Neurobiol..

[5] Reibel J. (2003). Tobacco and oral diseases. Update on the evidence, with recommendations. Med. Princ. Pract..

[6] Vellappally S., Fiala Z., Smejkalová J., Jacob V., Somanathan R. (2007). Smoking related systemic and oral diseases. Acta Med..

[7] Winn D.M. (2001). Tobacco use and oral disease. J. Dent. Educ..

[8] Hoare A., Soto C., Rojas-Celis V., Bravo D. (2019). Chronic Inflammation as a Link between Periodontitis and Carcinogenesis. Mediat. Inflamm..

[9] Kumar S. (2019). Evidence-Based Update on Diagnosis and Management of Gingivitis and Periodontitis. Dent. Clin..

[10] Geisinger M.L., Kaur M., Basma H. (2019). Nonsurgical Periodontal Therapy: A Review of Current Standards of Care and Innovations to Improve Gingival and Periodontal Health. Curr. Oral Health Rep..

[11] Graziani F., Karapetsa D., Alonso B., Herrera D. (2017). Nonsurgical and surgical treatment of periodontitis: How many options for one disease?. Periodontol. 2000.

[12] Tariq M., Iqbal Z., Ali J., Baboota S., Talegaonkar S., Ahmad Z., Sahni J.K. (2012). Treatment modalities and evaluation models for periodontitis. Int. J. Pharm. Investig..

[13] Arulselvan P., Fard M.T., Tan W.S., Gothai S., Fakurazi S., Norhaizan M.E., Kumar S.S. (2016). Role of Antioxidants and Natural Products in Inflammation. Oxidative Med. Cell. Longev..

[14] Hussain T., Tan B., Yin Y., Blachier F., Tossou M.C., Rahu N. (2016). Oxidative Stress and Inflammation: What Polyphenols Can Do for Us?. Oxidative Med. Cell. Longev..

[15] Bernstein N., Akram M., Daniyal M., Koltai H., Fridlender M., Gorelick J. (2018). Antiinflammatory Potential of Medicinal Plants: A Source for Therapeutic Secondary Metabolites. Adv. Agron..

[16] Rao Y.K., Fang S.H., Tzeng Y.M. (2008). Antiinflammatory activities of flavonoids and a triterpene caffeate isolated from Bauhinia variegata. Phytother. Res..

[17] Maas M., Deters A.M., Hensel A. (2011). Anti-inflammatory activity of *Eupatorium perfoliatum* L. extracts, eupafolin, and dimeric guaianolide via iNOS inhibitory activity and modulation of inflammation-related cytokines and chemokines. J. Ethnopharmacol..

[18] Hodge G., Hodge S., Han P. (2002). Allium sativum (garlic) suppresses leukocyte inflammatory cytokine production in vitro: Potential therapeutic use in the treatment of inflammatory bowel disease. Cytometry.

[19] Kim I.T., Park Y.M., Shin K.M., Ha J., Choi J., Jung H.J., Park H.J., Lee K.T. (2004). Anti-inflammatory and anti-nociceptive effects of the extract from Kalopanax pictus, Pueraria thunbergiana and Rhus verniciflua. J. Ethnopharmacol..

[20] Al-Ghamdi M.S. (2001). The anti-inflammatory, analgesic and antipyretic activity of Nigella sativa. J. Ethnopharmacol..

[21] Lu C.H., Li Y.Y., Li L.J., Liang L.Y., Shen Y.M. (2012). Anti-inflammatory activities of fractions from Geranium nepalense and related polyphenols. Drug Discov. Ther..

[22] Freitas A.P., Bitencourt F.S., Brito G.A., de Alencar N.M., Ribeiro R.A., Lima-Junior R.C., Ramos M.V., Vale M.L. (2012). Protein fraction of Calotropis procera latex protects against 5-fluorouracil-induced oral mucositis associated with downregulation of pivotal pro-inflammatory mediators. Naunyn Schmiedeberg’s Arch. Pharmacol..

[23] Anwar F., Latif S., Ashraf M., Gilani A.H. (2006). Moringa oleifera: A food plant with multiple medicinal uses. Phytother. Res..

[24] Leone A., Spada A., Battezzati A., Schiraldi A., Aristil J., Bertoli S. (2015). Cultivation, Genetic, Ethnopharmacology, Phytochemistry and Pharmacology of Moringa oleifera Leaves: An Overview. Int. J. Mol. Sci..

[25] Abd Rani N.Z., Husain K., Kumolosasi E. (2018). Moringa Genus: A Review of Phytochemistry and Pharmacology. Front. Pharmacol..

[26] Kou X., Li B., Olayanju J.B., Drake J.M., Chen N. (2018). Nutraceutical or Pharmacological Potential of Moringa oleifera Lam. Nutrients.

[27] de Oliveira R.G., Mahon C.P., Ascencio P.G., Ascencio S.D., Balogun S.O., de Oliveira Martins D.T. (2014). Evaluation of anti-inflammatory activity of hydroethanolic extract of Dilodendron bipinnatum Radlk. J. Ethnopharmacol..

[28] Hong Y.H., Chao W.W., Chen M.L., Lin B.F. (2009). Ethyl acetate extracts of alfalfa (*Medicago sativa* L.) sprouts inhibit lipopolysaccharide-induced inflammation in vitro and in vivo. J. Biomed. Sci..

[29] Jeong Y.H., Oh Y.C., Cho W.K., Lee B., Ma J.Y. (2016). Anti-Inflammatory Effects of Melandrii Herba Ethanol Extract via Inhibition of NF-kappaB and MAPK Signaling Pathways and Induction of HO-1 in RAW 264.7 Cells and Mouse Primary Macrophages. Molecules.

[30] Kooltheat N., Sranujit R.P., Chumark P., Potup P., Laytragoon-Lewin N., Usuwanthim K. (2014). An ethyl acetate fraction of Moringa oleifera Lam. Inhibits human macrophage cytokine production induced by cigarette smoke. Nutrients.

[31] Luetragoon T., Pankla Sranujit R., Noysang C., Thongsri Y., Potup P., Suphrom N., Nuengchamnong N., Usuwanthim K. (2020). Bioactive Compounds in Moringa oleifera Lam. Leaves Inhibit the Pro-Inflammatory Mediators in Lipopolysaccharide-Induced Human Monocyte-Derived Macrophages. Molecules.

[32] Kooltheat N., Sranujit R., Luetragoon T., Yuchat M., Adulyaritthikul P., Chaisomboon C., Potup P., Ferrante A., Usuwanthim K. (2017). Moringa oleifera Lam. leaves extract reduces human T-cell hyporesponsiveness and DNA damage induced by oxidative stress. Int. J. Res. Ayurveda Pharm..

[33] Arulselvan P., Tan W.S., Gothai S., Muniandy K., Fakurazi S., Esa N.M., Alarfaj A.A., Kumar S.S. (2016). Anti-Inflammatory Potential of Ethyl Acetate Fraction of Moringa oleifera in Downregulating the NF-kappaB Signaling Pathway in Lipopolysaccharide-Stimulated Macrophages. Molecules.

[34] Waterman C., Cheng D.M., Rojas-Silva P., Poulev A., Dreifus J., Lila M.A., Raskin I. (2014). Stable, water extractable isothiocyanates from Moringa oleifera leaves attenuate inflammation in vitro. Phytochemistry.

[35] Kim M.S., Kim S.H. (2011). Inhibitory effect of astragalin on expression of lipopolysaccharide-induced inflammatory mediators through NF-kappaB in macrophages. Arch. Pharm. Res..

[36] Li F., Liang D., Yang Z., Wang T., Wang W., Song X., Guo M., Zhou E., Li D., Cao Y. (2013). Astragalin suppresses inflammatory responses via down-regulation of NF-kappaB signaling pathway in lipopolysaccharide-induced mastitis in a murine model. Int. Immunopharmacol..

[37] Riaz A., Rasul A., Hussain G., Zahoor M.K., Jabeen F., Subhani Z., Younis T., Ali M., Sarfraz I., Selamoglu Z. (2018). Astragalin: A Bioactive Phytochemical with Potential Therapeutic Activities. Adv. Pharmacol. Sci..

[38] Engsuwan J., Waranuchb N., Limpeanchobc N., Ingkaninana B.K. (2017). HPLC methods for quality control of Moringa oleifera extract using isothiocyanates and astragalin as bioactive markers. ScienceAsia.

[39] Iwalewa E.O., Iwalewa O.J., Adeboye J.O. (2003). Analgesic, antipyretic, anti-inflammatory effects of methanol, chloroform and ether extracts of Vernonia cinerea less leaf. J. Ethnopharmacol..

[40] Joshi T., Pandey S.C., Maiti P., Tripathi M., Paliwal A., Nand M., Sharma P., Samant M., Pande V., Chandra S. (2021). Antimicrobial activity of methanolic extracts of Vernonia cinerea against Xanthomonas oryzae and identification of their compounds using in silico techniques. PLoS ONE.

[41] Yusoff S.F., Haron F.F., Tengku Muda Mohamed M., Asib N., Sakimin S.Z., Abu Kassim F., Ismail S.I. (2020). Antifungal Activity and Phytochemical Screening of Vernonia amygdalina Extract against Botrytis cinerea Causing Gray Mold Disease on Tomato Fruits. Biology.

[42] Puttarak P., Pornpanyanukul P., Meetam T., Bunditanukul K., Chaiyakunapruk N. (2018). Efficacy and safety of *Vernonia cinerea* (L.) Less. for smoking cessation: A systematic review and meta-analysis of randomized controlled trials. Complement. Ther. Med..

[43] Wongwiwatthananukit S., Benjanakaskul P., Songsak T., Suwanamajo S., Verachai V. (2009). Efficacy of Vernonia cinerea for smoking cessation. J. Health Res..

[44] Singh A., Saharan V.A., Kumawat I.C., Khatri A., Bhandari A. (2014). A pharmacognostical study of *Vernonia cinerea* Less (Asteraceae) and evaluation of anti-inflammatory and antibacterial activities of stem. Egypt Pharm. J..

[45] Ketsuwan N., Leelarungrayub J., Kothan S., Singhatong S. (2017). Antioxidant compounds and activities of the stem, flower, and leaf extracts of the anti-smoking Thai medicinal plant: Vernonia cinerea Less. Drug Des. Dev. Ther..

[46] Prasopthum A., Pouyfung P., Sarapusit S., Srisook E., Rongnoparut P. (2015). Inhibition effects of Vernonia cinerea active compounds against cytochrome P450 2A6 and human monoamine oxidases, possible targets for reduction of tobacco dependence. Drug Metab. Pharmacokinet..

[47] Toyang N.J., Verpoorte R. (2013). A review of the medicinal potentials of plants of the genus Vernonia (Asteraceae). J. Ethnopharmacol..

[48] Saraphanchotiwitthaya A., Sripalakit P. (2015). Anti-inflammatory activity of a Vernonia cinerea methanolic extract in vitro. ScienceAsia.

[49] Alara O.R., Abdurahman N.H., Ukaegbu C.I., Azhari N.H. (2018). Vernonia cinerea leaves as the source of phenolic compounds, antioxidants, and anti-diabetic activity using microwave-assisted extraction technique. Ind. Crops Prod..

[50] Sonibare M.A., Aremu O.T., Okorie P.N. (2016). Antioxidant and antimicrobial activities of solvent fractions of *Vernonia cinerea* (L.) Less leaf extract. Afr. Health Sci..

[51] Fitriana W.D., Ersam T., Shimizu K., Fatmawati S. (2016). Antioxidant Activity of Moringa oleifera Extracts. Indones. J. Chem..

[52] Xu Y.B., Chen G.L., Guo M.Q. (2019). Antioxidant and Anti-Inflammatory Activities of the Crude Extracts of Moringa oleifera from Kenya and Their Correlations with Flavonoids. Antioxidants.

[53] Sreelatha S., Padma P.R. (2009). Antioxidant activity and total phenolic content of Moringa oleifera leaves in two stages of maturity. Plant. Foods Hum. Nutr..

[54] Gurenlian J.R. (2009). Inflammation: The relationship between oral health and systemic disease. Dent. Assist..

[55] Elgamily H., Moussa A., Elboraey A., El-Sayed H., Al-Moghazy M., Abdalla A. (2016). Microbiological Assessment of Moringa Oleifera Extracts and Its Incorporation in Novel Dental Remedies against Some Oral Pathogens. Open Access Maced. J. Med. Sci..

[56] Jwa S.K. (2019). Efficacy of Moringa oleifera Leaf Extracts against Cariogenic Biofilm. Prev. Nutr. Food Sci..

[57] Nagarajappa R., Bhanushali N.V., Ramesh G., Bhanushali P.V., Aapaliya P., Pujara P. (2019). Antimicrobial Activity of Moringa Oleifera Extracts against Common Periodontal Pathogens: Potential Application in the Prevention and Treatment of Oral Diseases. Indian J. Public Health Res. Dev..

[58] Gupta M., Mazumder U.K., Manikandan L., Haldar P.K., Bhattacharya S., Kandar C.C. (2003). Antibacterial activity of Vernonia cinerea. Fitoterapia.

[59] Alshwerf A.O., Amin L.S., Ibrahim F., Youssef J. (2017). Protective Effect of Moringa oleifera Extract on Experimentally LPS-induced periodontitis. Int. J. Adv. Res..

[60] Sahrakary M., Nazemian V., Aghaloo M., Akbari A., Shadnoush M., Nasseri B., Zaringhalam J. (2017). Treatment by Moringa Oleifera Extract Can Reduce Gingival Inflammatory Cytokines in the Rat Periodontal Model. Physiol. Pharmacol..

[61] Mohanty M., Mohanty S., Bhuyan S.K., Bhuyan R. (2020). Phytoperspective of Moringa oleifera for oral health care: An innovative ethnomedicinal approach. Phytother. Res..

[62] Rozza A.L., Meira de Faria F., Souza Brito A.R., Pellizzon C.H. (2014). The gastroprotective effect of menthol: Involvement of anti-apoptotic, antioxidant and anti-inflammatory activities. PLoS ONE.

[63] Sun Z., Wang H., Wang J., Zhou L., Yang P. (2014). Chemical Composition and Anti-Inflammatory, Cytotoxic and Antioxidant Activities of Essential Oil from Leaves of Mentha piperita Grown in China. PLoS ONE.

[64] Martin B.J., Campbell P.M., Rees T.D., Buschang P.H. (2016). A randomized controlled trial evaluating antioxidant-essential oil gel as a treatment for gingivitis in orthodontic patients. Angle Orthod..

[65] Bakre A.G., Aderibigbe A.O., Ademowo O.G. (2013). Studies on neuropharmacological profile of ethanol extract of Moringa oleifera leaves in mice. J. Ethnopharmacol..

[66] Woo Shin J., Seol I.C., Son C.G. (2010). Interpretation of Animal Dose and Human Equivalent Dose for Drug Development. J. Korean Med..

[67] Aruna G., Jayachandra Reddy P., Prabhakaran V. (2012). Safety evaluation of ethanol extract of vernonia cinerea L. in experimental animals. Int. J. Pharm..

[68] Majekodunmi S.O. (2015). A Review on Lozenges. Am. J. Med. Med. Sci..

[69] Medina-Remon A., Barrionuevo-Gonzalez A., Zamora-Ros R., Andres-Lacueva C., Estruch R., Martinez-Gonzalez M.A., Diez-Espino J., Lamuela-Raventos R.M. (2009). Rapid Folin-Ciocalteu method using microtiter 96-well plate cartridges for solid phase extraction to assess urinary total phenolic compounds, as a biomarker of total polyphenols intake. Anal. Chim. Acta.

[70] Clarke G., Ting K.N., Wiart C., Fry J. (2013). High Correlation of 2,2-diphenyl-1-picrylhydrazyl (DPPH) Radical Scavenging, Ferric Reducing Activity Potential and Total Phenolics Content Indicates Redundancy in Use of All Three Assays to Screen for Antioxidant Activity of Extracts of Plants from the Malaysian Rainforest. Antioxidants.

[71] Zheng J.Z., Li Y., Lin T., Estrada A., Lu X., Feng C. (2017). Sample Size Calculations for Comparing Groups with Continuous Outcomes. Shanghai Arch. Psychiatry.

[72] Sonis S.T., Eilers J.P., Epstein J.B., LeVeque F.G., Liggett W.H., Mulagha M.T., Peterson D.E., Rose A.H., Schubert M.M., Spijkervet F.K. (1999). Validation of a new scoring system for the assessment of clinical trial research of oral mucositis induced by radiation or chemotherapy. Mucositis Study Group. Cancer.

[73] Tobias G., Spanier A.B. (2020). Modified Gingival Index (MGI) Classification Using Dental Selfies. Appl. Sci..

